# Optimum rescheduling of water networks for batch processes using a goal programming technique

**DOI:** 10.1038/s41598-023-49070-9

**Published:** 2023-12-14

**Authors:** Fatma M. Ayyad, Walaa M. Shehata, Ahmed A. Bhran, Abeer M. Shoaib

**Affiliations:** 1 Department of Petroleum Refining and Petrochemical Engineering, Faculty of Petroleum and Mining Engineering, Suez University, P.O. Box: 43221, Suez, Egypt; 2 Chemical Engineering Department, College of Engineering, Imam Mohammad Ibn Saud Islamic University, 11432 Riyadh, Saudi Arabia

**Keywords:** Environmental sciences, Engineering, Mathematics and computing

## Abstract

Batch processes are relevant to a wide variety of industries in chemical processes. In batch operations, water sources are almost not directly reused/recycled in process sinks without considering time constraints and storage tanks. However, storage tanks are usually expensive and thus a cost-effective water system has to be synthesized. Rescheduling the water network can contribute to reducing the cost of storage tanks by reducing their number and capacity. In the current research work, a goal programming is used to reschedule the water network in batch processes considering the time and storage tanks. A Mixed Integer Non-Linear Program model is introduced using the Lingo optimization program. This model is used to optimize multiple objectives, which are freshwater usage, wastewater discharge, the number and capacity of tanks, the degree of shifting streams, and the total cost of the water network. Three case studies are presented in this study to demonstrate the effectiveness of the proposed procedure, considering both single and multi-contaminants problems. The results of the first case study show a reduction in the network cost and the freshwater flowrate by 26.4% and 42%, respectively. Regarding the rescheduled water network results of the second case study, the cost is reduced by 24.6% and the freshwater flowrate is decreased by 21.8% with no requirement of storage tanks. The third case study highlights the model’s applicability to multi-contaminants problem, revealing a 15.1% cost reduction and a 25.7% decrease in freshwater flow.

## Introduction

Water is a crucial natural resource in industrial processes. The increased consumption of natural resources, especially freshwater, is due to socioeconomic developments that have resulted in freshwater shortage^1^. Water recovery by direct reuse or recycling can be considered one of the most effective ways to minimize the amount of freshwater used in batch processes in the chemical industry.

Batch processes have got a lot of interest from chemical industries because of their flexibility and adaptability for producing specialized low-volume products^2^. Scheduling of batch process reduces both freshwater consumption and wastewater discharge; hence, for sub-optimal results, rescheduling the batch process is required. The key difference between scheduling and rescheduling lies in how disturbances could be handled, such as unexpected events or changes in priorities. Scheduling seeks to achieve global optimality under normal conditions, whereas rescheduling is designed to address the impact of disturbances on the previously optimized plan.

Rescheduling could be defined as a scheduling modification process that requires adjusting the time period for each process while maintaining the concentration constraints. Two different strategies for rescheduling are rescheduling by shifting and optimization. Algorithms are used in the shifting technique, but the rule-based deterministic method is used in rescheduling by optimization^3^. In batch processing systems, storage tanks can be used to store water and dispatch it when it is needed in subsequent batches in order to enhance water recovery in the plant. Rescheduling batch processes is necessary to reduce both the number and size of the required water storage tanks, thereby increasing the chances of direct reuse and recycling. As a result, having an effective rescheduling operations strategy is critical^4^.

Rescheduling or reactive scheduling of batch chemical processes has been presented in much research; Kemp and Macdonald were the first who demonstrated rescheduling for batch heat exchanger networks. However, they did not demonstrate a systematic methodology to the synthesis of these networks^5^. on the other hands, Obeng and Ashton used a time-dependent chart to guide the rescheduling process, which determines the time interval for water intake and discharge, which is based on trial-and-error efforts^6^, though, this method does not guarantee the optimality Kemp and Deakin then showed how the cascade analysis could be used to systematically identify rescheduling possibilities and categorizing them into four types; Type 1 involves rescheduling without altering durations, optimizing mass transfer in parallel operations. Type 2 focuses on adjusting the relative timing within a single batch process cycle while maintaining durations. Type 3 entails changing stream durations by adjusting flowrates while preserving start or finish times. Lastly, Type 4 encompasses the modification of both timing and duration, resulting in the stream occurring at a different point within the batch operation^7^. Although Kemp and Deakin method is considered a good guide to rescheduling process, it is considered as sequential method, which could not result in global optimal solution.

Some mathematical-based optimization approaches for batch networks have been introduced to solve the problem of rescheduling; Kondili et al. used the State Network Task (SNT) concept in discrete time notations to provide a comprehensive framework based on mixed integer linear programming (MILP) to deal with multipurpose batch plants problems. However, this approach required a lot of computational work^8^. Ferrer-Nadal et al.^9^ presented a rescheduling framework using MILP, which includes recipe flexibility as an alternative for rescheduling, but their method could result in high violation to the process operation, which does not guarantee the reliability in application and does not take into consideration the total cost. To overcome this disadvantage. Shoaib et al. focused on developing cost-effective batch water networks that incorporate a range of process sources with freshwater, which are then blended, stored, and distributed to process sinks. A three-stage hierarchical approach is proposed to tackle the problem’s complexity. First, global targets are set using linear program to optimize water usage, recycling, and wastewater discharge without specifying the network. Then, a network with the minimum number of tanks is designed via a mixed-integer linear program, simplifying it for global solvability. Finally, the network’s configuration is streamlined by minimizing interconnections^4^. However, Shoaib et al. methodology is considered as sequential method and could not take into consideration all the affecting variable simultaneously. Chen and Lee presented the graphical technique that was used to examine the water reuse potential and determine the minimum freshwater consumption and wastewater generation^10^, but their work is limited for very simple problems due to difficulty of applying graphical methods on nearly high number of streams. In addition, it could not manipulate various parameters such as the required storage tanks or the cost. Kim presented a novel systematic targeting and design strategy to maximize water reuse in discontinuous water systems by establishing upper and lower-bound targets for reusing water at various time intervals^11^ though, this work focused only on one parameter, which is the amount of reused or recycled water.

A mathematical optimization model for the synthesis of an inter-plant water network (IPWN) has been proposed by Lee et al.^12^ with process units operating in both continuous and batch modes. Lee et al.^13^ extended the previous research on freshwater minimization to consider minimum storage capacity and interconnections using a four-stage mathematical model. However, the integration between the required storage capacity and freshwater usage and their integrated effect on the total cost is not taken into consideration in their work to overcome this disadvantage, Chaturvedi et al.^14^ aimed at reducing the operating cost of multiple water resources in rescheduling, which can lead to a decrease in the operating cost of individual water resource system; Nevertheless, number and capacity of storage tanks have not been taken into consideration in their work.

A mathematical approach for simultaneous batch production optimization and wastewater minimization in fixed mass load situations was developed by Adekola and Majozi^15^. The amount of wastewater produced can be decreased by analyzing the sequence of tasks inside a unit. They considered both sequence-dependent setup costs and a goal of profit maximization. With the aim of representing the fundamental properties of water flows, Pulluru and AKKerman^16^ developed a mathematical programming-based water-integrated scheduling system. Their evaluation of water reuse and regeneration technologies for typical industrial settings is represented as a demonstration of the use of the proposed technique in this work. Additionally, they simulated water quality using a realistic and general classification scheme in order to efficiently integrate water reuse and treatment options. Li et al. proposed a heuristic approach that employs the concept of concentration potential for designing batch water-using networks that contain multiple contaminants along with regeneration units. The regenerated stream(s) can be incorporated into the reuse-only network to create a water-using system with regeneration units. The design process of the network operating in a single batch mode considers time as the primary factor, with concentration potentials being of secondary importance^17^. Cansino-Loeza et al. investigated a multi-objective optimization model designed to achieve the ideal water-energy nexus for a residential complex. They provided an optimization formulation to meet the needs of a self-sufficient community for food, energy, and water. The authors suggested a mixed-integer linear programming model that simultaneously considers three objectives: emissions, freshwater consumption, and profits. Specifically, the model aims to minimize emissions and freshwater consumption while maximizing profits. The proposed model thus offers a comprehensive approach to designing sustainable and profitable water-energy systems for residential communities^18^. A new integrated model and algorithm have been added by Ősz et al. to the S-graph scheduling framework, aiming to maximize batch process water reuse within a specified time horizon. This model enforces the constraint that only a single water source can be reused for each sink. Tasks are assigned a predefined time horizon defined by their duration, rather than starting and ending times^19^.

Among these research works, no simultaneous technique has been introduced to determine the best rescheduling configuration that aims to minimize schedule violation and introduce the required time-shifting in processes, while approaching minimum freshwater usage, minimum wastewater discharge, minimum tank capacity and minimum cost.

Goal programming has been used to handle large-scale multi-criteria decision-making challenges. It is a decision-making technique that was created to solve multi-objective problems for the best solution. It uses linear programming to obtain the optimal solution for a set of linearly expressed constraints on a single-dimensional or multi-dimensional objective function^20^. In this study, a new mathematical optimization model based on goal programming is developed to produce a low-cost batch water network with a rescheduling strategy aiming to maximize water recovery while minimizing the violation from the existing schedule. This approach synthesizes and reschedules the batch water process by the LINGO optimization program. A novel mixed integer nonlinear program (MINLP) multi-objective optimization is presented.

## Problem statement

This study focuses on the problem of rescheduling a batch water network. A single key contaminant is considered. The problem could be formulated as follows:

- A set of batch process water sinks {SKj = 1, 2, …, Nj} is given; with each sink SK_j_ requiring a specific flowrate (F_SKj_) and having a maximum allowable inlet impurity concentration (C_SKj_).
- A set of batch process water sources {SR_i_ = 1, 2, …, Ni} is also given; where each source SRi has a specific flowrate (F_SRi_) and impurity concentration level of (C_SRi)_. The water sources can be stored in tanks and subsequently reused/recycled to the sinks as long as they meet the sinks’ flow and composition requirements. A source of fresh (external) water should be available to satisfy the water sink’s requirements.

The following data should also be given:

- Initial and final time for each source; T_i(SRi)_ and T_f(SRi)_, respectively.
- Initial and final time for each sink; T_i(Skj)_ and T_f(Skj),_ respectively.

This work aims to introduce the best rescheduling scheme with minimum required time-shift, resulting in a cost-effective batch water network.

## Methodology

**Figure 1:**
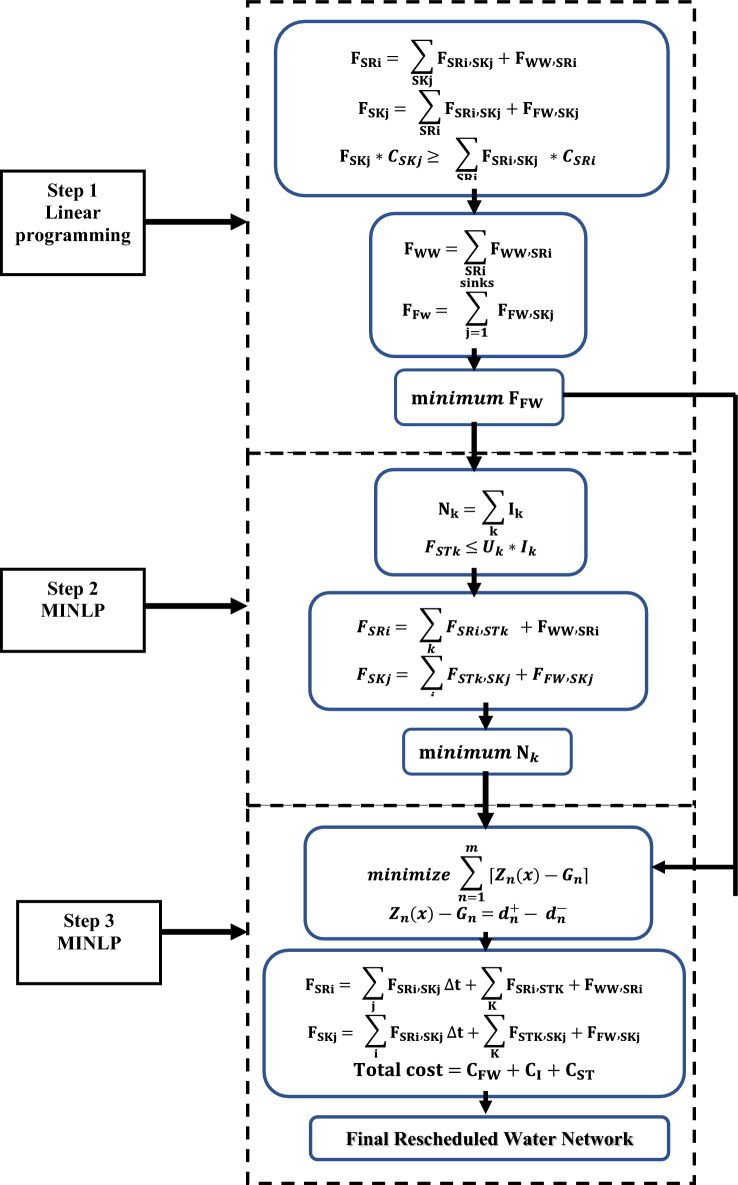
Flowchart for the proposed model.

To get the best rescheduling scheme by goal programming, a multi-objective function model is introduced. This model aims to achieve multiple objectives, including minimizing freshwater requirements, minimizing wastewater discharge, optimizing the number of tanks, and minimizing the degree of stream shifting, through goal programming to obtain the best rescheduling scheme. The proposed approach comprises three steps. The first step involves identifying targets for the minimum freshwater supply and the minimum wastewater discharge. Regarding the second step, the objective is to minimize the number of water storage tanks in order to reduce the complexity of the batch network. In the last stage, the goal programming model is introduced by using the results from the first and second steps to determine the degree of streams’ shifting; consequently, the minimum total cost is achieved. The three steps are discussed in detail in the following paragraphs and the flowchart for the proposed model is shown in the Fig. 1.

### Step 1: Minimizing freshwater requirement

**Figure 2:**
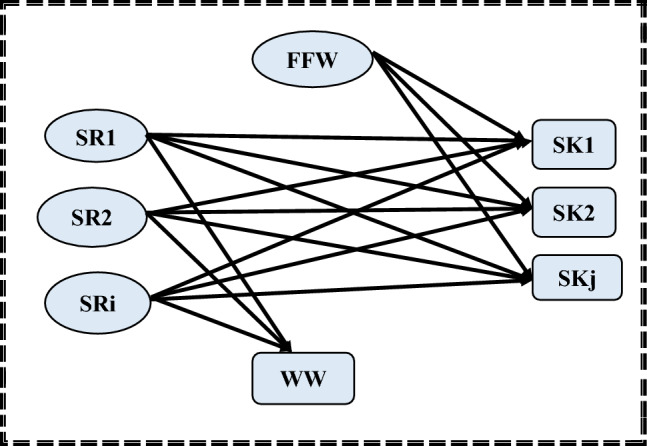
A simplified superstructure for minimizing freshwater requirement (step 1).

In the first step of the proposed approach, the aim is to determine the minimum flowrate required for freshwater supply, while minimizing the amount of wastewater discharge in the investigated batch water network. Water storage tanks are not considered in this step. Therefore, as shown in Fig. 2, the problem is formulated by assigning water sources directly to sinks through mixing points, without the need for storage tanks. As a result, the transshipment model can be simplified to a transportation formulation that minimizes the total amount of freshwater transported to the water system (FFW). This could be accomplished by employing the following set of equations:

1 $$ {\text{Minimize }}\,{\text{F}}_{{{\text{FW}}}} $$

2 $$ {\text{Subjected}}\,{\text{ to}} \, {\text{F}}_{{{\text{Fw}}}} = \sum\nolimits_{{{\text{j}} = 1}}^{{{\text{sinks}}}} {{\text{F}}_{{\text{FW,SKj}}} } $$

3 $$ {\text{F}}_{{{\text{SRi}}}} = \sum\nolimits_{{{\text{SKj}}}} {{\text{F}}_{{\text{SRi,SKj}}} + {\text{F}}_{{\text{WW,SRi}}} } { }\quad { } = 1,2,3, \ldots {\text{N}}_{{\text{i}}} $$

4 $$ {\text{F}}_{{{\text{SKj}}}} = \sum\nolimits_{{{\text{SRi}}}} {{\text{F}}_{{\text{SRi,SKj}}} } { } + {\text{F}}_{{\text{FW,SKj }}} \quad {\text{j}} = 1,2,3, \ldots {\text{N}}_{{\text{j}}} $$

5 $$ {\text{F}}_{{{\text{WW}}}} = \sum\nolimits_{{{\text{SRi}}}} {{\text{F}}_{{\text{WW,SRi}}} } $$

6 $$ {\text{F}}_{{{\text{SKj}}}} {*}C_{SKj} \ge { }\sum\nolimits_{{{\text{SRi}}}} {{\text{F}}_{{{\text{SRi}}}} ,_{{{\text{SKj}}}} {*}C_{SRi} } \quad j = 1,2,3 \ldots N_{j} $$

The initial set of equations states that a batch water network’s total freshwater flow is equal to the sum of the freshwater supplied to each water sink (F_FW,SKj_). The subsequent set of equations illustrates the flow balance for each water source. Specifically, the water available from each source (F_SRi_) is allocated to a number of water sinks, while any remaining portion is discharged as wastewater (F_WW,SRi_). The rest of the equations specify concentration constraints.

### Step 2: Obtaining the minimum number of storage tanks.

Once the minimal freshwater flow target is established as discussed in the first stage, the second step is to determine the minimum number of storage tanks required to achieve the minimal freshwater flow target. The model formulation for the second stage is illustrated in the following set of equations:

7 $$ {\text{Minimize }}\,{\text{N}}_{{\text{k}}} $$

where N_k_ represents the total number of tanks

8 $$ {\text{N}}_{{\text{k}}} = \sum\nolimits_{{\text{k}}} {{\text{I}}_{{\text{k}}} } $$

Equation (9) defines the binary variable I_k_, which indicates whether the storage tank (k) exists or not.

9 $$ F_{STk} \le U_{k} *I_{k} $$

10 $$ F_{SRi} = { }\mathop \sum \limits_{k} F_{{\text{SRi,STk}}} { } + {\text{F}}_{{\text{WW,SRi}}} \quad i = 1,2,3, \ldots N_{i} $$

11 $$ F_{STk} = \sum\nolimits_{i} {F_{SRi,STk} } \quad k = 1,2,3, \ldots N_{k} $$

12 $$ F_{STk} {*}C_{STk} = { }\mathop \sum \limits_{i} F_{SRi,STk} {*}C_{SRi} \quad i = 1,2,3, \ldots N_{i} $$

13 $$ F_{SKj} = \sum\nolimits_{j} {F_{STk,SKj} } { } + F_{{FW,SKj{ }}} \quad j = 1,2,3, \ldots N_{j} $$

14 $$ F_{SKj} {*}C_{SKj} \ge { }\mathop \sum \limits_{j} F_{STk,SKj} {*}C_{STk} { }\quad j = 1,2,3 \ldots N_{j} $$

The parameter U_k_ represents the maximum permitted capacity of tank (ST_K_) in the considered batch water network. If the storage tank capacity (F_STk_) is zero, the value for I_k_ may be set to zero. However, since the objective function aims to minimize the number of tanks, the optimal solution is obtained when I_k_ value equals zero, indicating that the ST_k_ of the storage tank should be eliminated. On the other hand, if the storage tank capacity (F_STk_) is greater than zero, the value of I_k_ must be set to 1, indicating the presence of the storage tank capacity parameter (STk) in the batch water network under consideration.

The constraints described in Eq. (10) correspond to the mass balance surrounding each water source SRi where the distribution of water available from these sources among the storage tanks (each stream has a flow of F_SRi,STk_). Equations (11) and (12) define the water flow (F_STk_) and impurity balances of the water storage tanks. Equations (13) and (14) specify that each water sink (SKj) is supplied by fresh water (F_FWskj_) and/or storage water, and a mixture of these sources should satisfy the impurity constraints of each sink.

### Step 3: Obtaining the best rescheduled water network scheme

As discussed before this third stage considers the applying of the goal programming model to achieve the optimum water network with minimum total cost. It involves three main types of analysis: first, determining the resources required to achieve a set of objectives; second, determining the extent to which the goals can be met given the available resources; and third, identifying the most satisfactory solution under various resource constraints and goal priorities. By applying goal programming, decision-makers can effectively handle trade-offs between objectives and achieve a desirable outcome that meets their specific needs and preferences.

Equation (15) presents the objective function of this third step as shown below:

15 $$ minimize\sum\nolimits_{n = 1}^{m} {Z_{n} \left( x \right) - G_{n} } { } $$

where Z_n_ is a linear function of nth goal, G_n_ is the aspiration level of nth goal and m is the total number of goals.

An aspiration level represents the desired level of attainment for a particular objective or goal, and it is often expressed as a numerical value or a range. On the other hand, a goal is a desired outcome or objective that a decision maker seeks to achieve, and it typically includes an objective function and an aspiration level. Both of the objective function and aspiration level define the criteria that the decision maker will use to evaluate potential options or courses of action.

16 $$ Z_{n} \left( x \right) - G_{n} = d_{n}^{ + } - { }d_{n}^{ - } \quad {\text{d}}_{{\text{n}}}^{ + } ,{\text{ d}}_{{\text{n}}}^{ - } \ge 0 $$

where ${\mathrm{d}}_{\mathrm{n}}^{+}, {\mathrm{d}}_{\mathrm{n}}^{-}$ represent under and over deviations of nth goals respectively^18^.

#### Mass balance around sources

Mass balance for sources is shown by Eq. (17).

17 $$ {\text{F}}_{{{\text{SRi}}}} = { }\sum\nolimits_{{\text{j}}} {{\text{F}}_{{\text{SRi,SKj}}} } \Delta {\text{t}} + \sum\nolimits_{{\text{K}}} {{\text{F}}_{{\text{SRi,STK}}} } + {\text{F}}_{{\text{WW,SRi}}} ,{ }\quad {\text{j}} = 1,2,3, \ldots {\text{N}}_{{\text{i}}} $$

where ∆t is the time period during which overlapping between SR_i_ and SK_j_ is occurred. Each source is either provided to sink SKj at the same time, stored for reuse/recycling, or released as wastewater.

#### Mass balance around sinks

Equation 18 presents the mass balance around sinks

18 $$ {\text{F}}_{{{\text{SKj}}}} = \sum\nolimits_{{\text{i}}} {{\text{F}}_{{\text{SRi,SKj}}} } { }\Delta {\text{t}} + \sum\nolimits_{{\text{K}}} {{\text{F}}_{{\text{STK,SKj}}} } + {\text{F}}_{{\text{FW,SKj}}} \quad {\text{K}} = 1,2, \ldots {\text{N}}_{{\text{K}}} $$

It should be noticed that each sink is either provided by a source SRi at the same time interval, water stored in storage tanks, or by an external freshwater to meet its flowrate requirement.

#### Mass balance around storage tanks

19 $$ {\text{F}}_{{{\text{STk}}}} = \sum\nolimits_{{\text{i}}} {{\text{F}}_{{\text{SRi,STk}}} } { } $$

20 $$ {\text{F}}_{{{\text{STk}}}} = { }\sum\nolimits_{{\text{j}}} {{\text{F}}_{{\text{STk,SKj}}} } $$

Equations (19) and (20) used to determine the total water flow into and out of a storage tank (FSTk). In Eq. (19), we calculate the total water flow into the storage tank (F_STk_) by aggregating the contributions from each water source (F_SRi,STk_) connected to the tank. Essentially, this equation sums up how much water each source adds to the tank.

Subsequently, in Eq. (20), we ascertain how much water from the storage tank (F_STk_) is distributed to various water sinks (SK_j_). This is achieved by summing the individual contributions from the tank to each sink (F_STk,SKj_). In other words, it calculates how the total flow from the tank is divided among different sinks.

#### Concentration constraints

Concentration constraints are described by Eqs. (21) and (22).

21 $$ {\text{F}}_{{{\text{STk}}}} {\text{*C}}_{{{\text{STk}}}} = \sum\nolimits_{{\text{i}}} {{\text{F}}_{{\text{SRi,STk}}} } {\text{ *C}}_{{{\text{SRi}}}} $$

22 $$ {\text{F}}_{{{\text{SKj}}}} {\text{*C}}_{{{\text{SKj}}}} \ge \sum\nolimits_{{\text{i}}} {{\text{F}}_{{\text{SRi,SKj}}} } { }\Delta {\text{t*C}}_{{{\text{SRi}}}} + \sum\nolimits_{{\text{k }}} {{\text{F}}_{{\text{STk,SKj}}} {\text{*C}}_{{{\text{STk}}}} } $$

where i = 1,2,3…N_i_ j = 1,2,3,…N_j_ k = 1,2,3,…N_k_.

#### Obtaining the minimum total cost

The total optimum cost for the investigated water network can be achieved by the following equations:

23 $$ {\text{Total }}\,{\text{cost}} = {\text{C}}_{{{\text{FW}}}} + {\text{C}}_{{\text{I}}} + {\text{C}}_{{{\text{ST}}}} $$

24 $$ {\text{C}}_{{{\text{ST}}}} = {\text{r}}.{\text{ST}} + {\text{s}} $$

where C_FW_ is the cost of freshwater, C_I_ is the cost of interception, and C_ST_ is the cost of storage tanks.

Kim and Smith used Eq. (24) to estimate the cost of storage tanks^21^. Regarding this equation, the slope of this linear relationship is referred to as r, and the interception is referred to as s. According to Kim and Smith, the cost parameters r and s for a carbon steel storage tank in mid-1980 were 116.95 and 10,142.16, respectively^21^. The Marshal1 and Swift (M and S) cost index is used to update the cost parameters. M and S for 1980 and 2017 were found to be 813 and 1593.7, respectively^22^.

In the final step of the process, a time-dependent chart is generated to serve as a valuable tool for problem formulation and analysis. This chart is essentially a dynamic structural graph that evolves over time, with edges representing connections or relationships between different elements within the system. These edges become active based on sequences of time-dependent events or conditions and may feature dynamic weights that change to reflect evolving degrees of influence or importance between the connected components. This time-dependent chart offers a visual representation of how information and interactions flow within the system, aiding decision-makers in understanding and addressing complex, time-sensitive problems^23^. The primary purpose of the chart is to guide the rescheduling process, which involves determining the optimal timing for water intake and discharge. The starting and ending times for each source and sink can be used as aspiration levels in this process. Additionally, the time-dependent chart is used in this study to calculate the cost, freshwater flowrate, and wastewater flowrate of the process prior to any rescheduling. Equations (17–24) are utilized for this purpose. These calculations provide important information about the current state and efficiency of the process, which can inform the decision-making process for the water network rescheduling.

## Case studies

For the current research work, two case studies are considered for applying goal programming to obtain the best rescheduling scheme of the investigated water networks. These two case studies are described in the following two subsections.

### Case study 1

The first case study used in this work is adapted from Almato ´ et al., Li and Chang, Shoaib et al.^4,24,25^. Table 1 shows the water stream data for this case study.

**Table 1:** Water data for the case study 1.

Water sinks SK_j_	Flow, F_SKj_ (m^3^)	Concentration, C_SKj_ (ppm)	Starting time, T^s^ (h)	Ending time, T^F^ (h)
SK1	20	0	0.5	2.5
SK2	20	6	5	7
SK3	20	15	9.5	11.5
SK4	16	5	17	19
SK5	20	7	6	8

Water sources, SR_i_	Flow, F_SKj_ (m^3^)	Concentration, C_SRi_ (ppm)	Starting time, T^s^ (h)	Ending time, T^f^ (h)
SR1	20	5	2.5	4.5
SR2	20	14	7	9
SR3	20	20	11.5	13.5
SR4	8	25	17	19
SR5	16	10	10.5	14.5

A hybrid of mass transfer and non-mass transfer-based water-using processes is included in this case study. The water sinks and sources of processes 1, 2, and 3 are SK1, SR1, SK2, SR2, and SK3, SR3, respectively. Because of the uniform flow at the input and outflow of each individual process, these processes are classified as mass transfer processes^4^. However, processes 4 and 5 are basically non-mass transfer-based processes. This can be attributed to their distinct intake and exit flowrates. In addition, each operation’s start time (t^s^) and finish time (t^f^) are specified. The quantities of freshwater and wastewater for this case study are 96 m^3^ and 84 m^3^, respectively, without water reuse. The initial stage in constructing the batch water network is to target minimum freshwater flow and wastewater discharge using the procedure described in step 1. The minimum number of tanks is calculated in the second step using 180 m^3^ as the maximum size of the water storage tanks. In the third step, Goal programming are applied as described by Eqs. (15–24). Cost data required for this stage could be taken as follows: annual operating time for batch processes is 8000 h, while freshwater (zero content of the pollutant) with cost of $1/kg can be used as needed.

### Case study 2

The second case study in this work is taken from Foo et al.^26^. The limiting data for this case study is listed in Table 2. It should be noticed that the annual operating time used for these batch processes is 8000 h. The freshwater (with no contamination of pollutant) used to supply the process sinks costs $1/kg.

**Table 2:** Water data for the case study 2.

Water sinks, SK_j_	Flow, (ton)	Concentration, C_SKj_, (ppm)	Starting time, T^s^ (h)	Ending time, T^F^ (h)
SK1	50	20	0	2
SK2	100	50	2	3
SK3	80	100	1.5	4
SK4	70	200	3	5

Water sources, SR_i_	Flow, (ton)	Concentration, C_Sri_, (ppm)	Starting time, T^s^ (h)	Ending time, T^f^ (h)
SR1	50	50	4	6
SR2	100	100	4	5
SR3	70	150	6	7
SR4	60	250	7	10

For this case study, the flow rates of freshwater and wastewater are 300 and 280 tons, respectively, and there is no water reuse. The minimum number of tanks is obtained in the second step based on a water storage tanks’ maximum size of 580 ton.

### Case study 3

The third case study in this paper is adapted from Foo et al.^27^ to address a multi-contaminants problem. Table 3 provides the key data for this case study. It should be noticed that each source or sink stream involves two contaminants, each with its own concentration. The annual operating time for these batch processes is 8000 h. The freshwater used to supply the process sinks, with no contamination of pollutants, costs $1 per kilogram.

**Table 3:** Water data for the case study 3.

Water sinks, SK_j_	Flow, (ton)	Contaminants	Concentration, C_SKj_, (ppm)	Starting time, T^s^ (h)	Ending time, T^F^ (h)
SK1	50	A	20	0	2
		B	11		
SK2	100	A	50	2	3
		B	30		
SK3	80	A	100	1.5	4
		B	60		
SK4	70	A	200	3	5
		B	140		

Water sources, SR_i_	Flow, (ton)	Contaminants	Concentration, C_Sri_, (ppm)	Starting time, T^s^ (h)	Ending time, T^f^ (h)
SR1	50	A	50	4	6
		B	30		
SR2	100	A	100	4	5
		B	50		
SR3	70	A	150	6	7
		B	120		
SR4	60	A	250	7	10
		B	240		

## Results and discussion

For the case study 1, a global solution is obtained by solving the linear programming (LP) equations as discussed in step 1. There are 17 constraints and 37 linear variables in the entire mathematical formulation for this step. According to the superstructure solution, the minimum freshwater flow and wastewater discharge are 35 and 23 m^3^, respectively.

**Figure 3:**
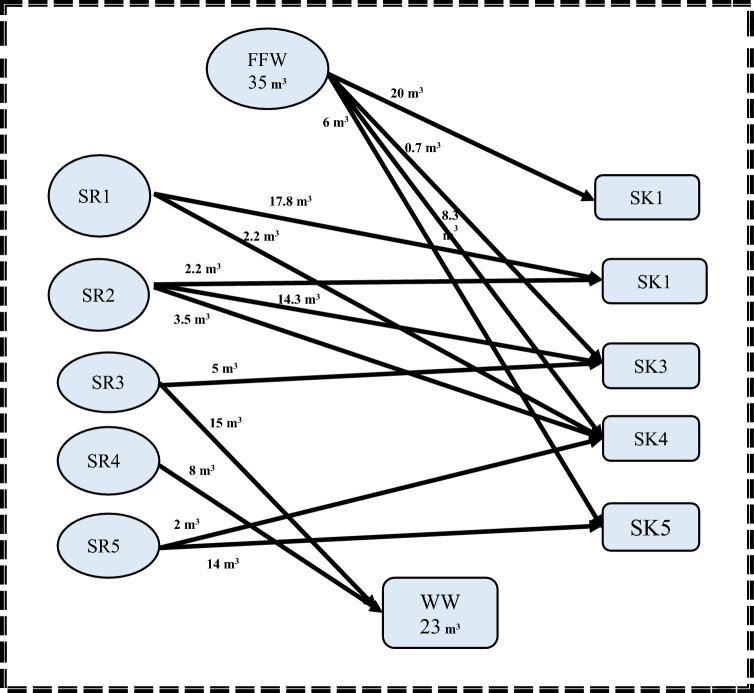
Water network with minimum freshwater for case study 1.

**Figure 4:**
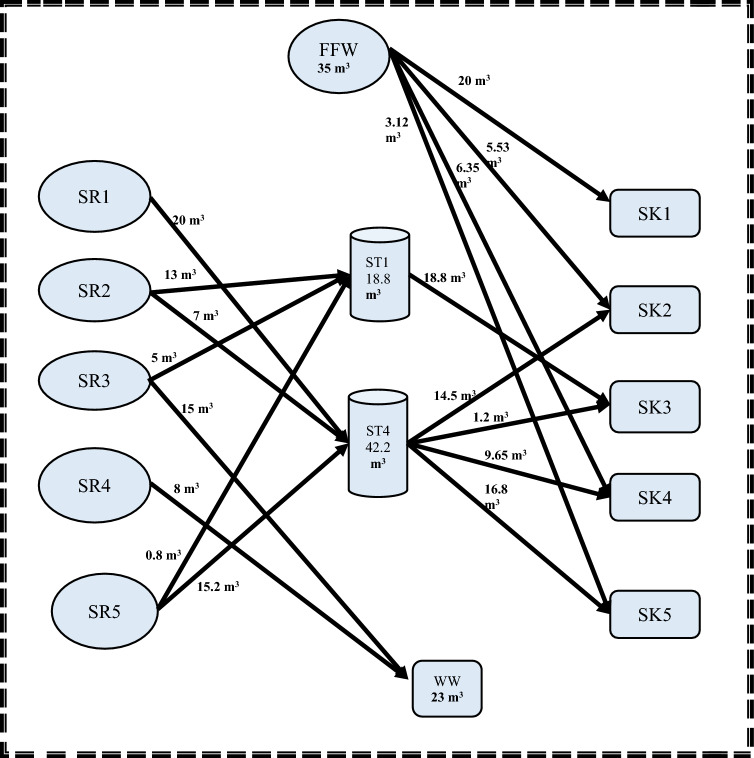
Water network with minimum number of tanks for case study 1.

Regarding the second step, the MINLP mathematical formulation has 78 variables, 35 non-linear variables, and 5 integers. Additionally, the elapsed runtime for this step was 14 s. Storage 1 (FST_1_ = 18.80218 m^3^; CST_1_ = 15.427 ppm) and Storage 4 (FST_4_ = 42.1978 m^3^; CST_4_ = 8.2926 ppm) are the two minimum units of required water storage tanks, as depicted. It is worth noting that the water network meets the minimum fresh and wastewater flow targets established in the first stage. These results matched well with results introduced by Shoaib et al.^4^. The mathematical results for the first and second stages are shown in Figs. 3 and 4, respectively.

Before applying the third step, it is important to calculate the cost, flow of freshwater and number and capacity of tanks before rescheduling. These calculations depend on the time dependent chart and the equations from17 to 24 with consideration of the starting and ending time for each source/sink.

**Figure 5:**
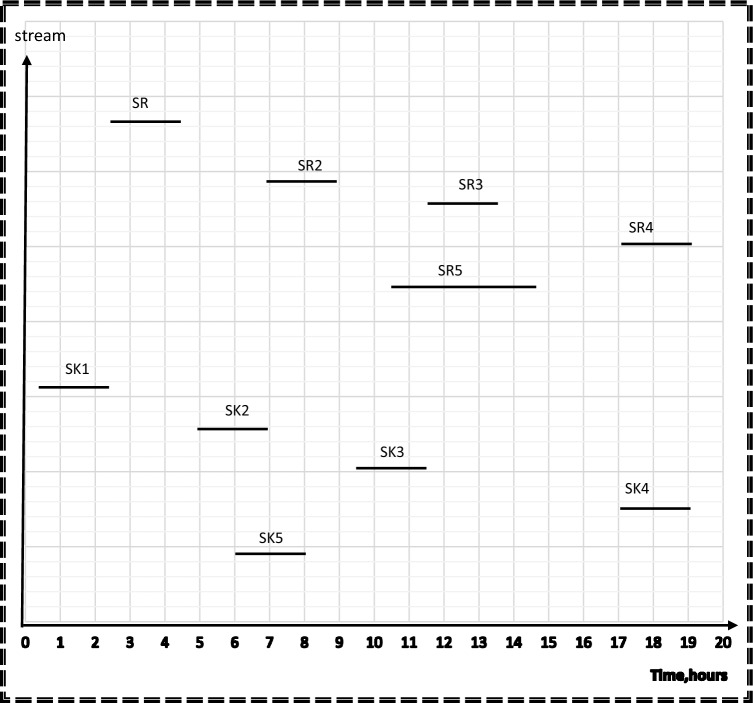
Time dependent chart for case study 1 before rescheduling.

In step 3, the results from step 1 and 2 will be used as aspiration levels of Z_n_(x) for freshwater and number of tanks in the objective function. Time dependent chart represents sources and sinks in their corresponding time intervals as continuous lines as shown in Fig. 5. This Figure is used as a guidance to calculate the cost, freshwater flowrate, and wastewater flowrate of the process before rescheduling. Minimum freshwater from step 1, which is 35 m^3^, is used as the aspiration level for Z_1_(x) for freshwater function. A mixed integer nonlinear program (MINLP) was run on Hyper LINGO (version API 12.0.3977), using Eqs. (1–24). The computation was performed on a PC with a 2.40 GHz processor and 8 GB of RAM. The goal programming model has 60 variables, 29 non-linear variables, and 57 constraints in its mathematical formulation. The computational process for this model, which includes solving and obtaining results, took 26 s.

A goal programming model in LINGO software is capable to solve multi objective functions in one program which are freshwater, wastewater, minimum number of water storage tanks, starting and ending time for sources and sinks, and minimum cost.

Comparing the results of water network after rescheduling with that obtained before rescheduling for case study 1 as shown in Table 4, the flowrate of freshwater has been decreased from 64.078 to 37.142 m^3^ by reduction percent of 42%. Additionally, the wastewater flowrate has been decreased from 52.078 to 25.141 m^3^. It is worth noting that the number of storage tanks required is reduced from two to one. Furthermore, the capacity of the required storage tank has been reduced to 26 m^3^ instead of a total size of 32 m^3^. The economic study showed that the total cost required for the network before rescheduling is $ 85,401.6, while the cost for the resulted network after rescheduling is reduced to $62,836.3 by reduction percent of 26.4%. The reduction in the total cost also be attributed to the decreasing of storage tank capacity by 18.8%.

**Table 4:** Comparison of the results before and after rescheduling for case 1.

Parameters	Before rescheduling	After rescheduling	Percentage of decreasing (%)
Freshwater	64.078 m^3^	37.14 m^3^	42
wastewater	52.078 m^3^	25.14 m^3^	51.7
Number of tanks	2	1	50
Capacity of tanks	32 m^3^	26 m^3^	18.8
Cost	85,401.6 $	62,836.26 $	26.4

**Figure 6:**
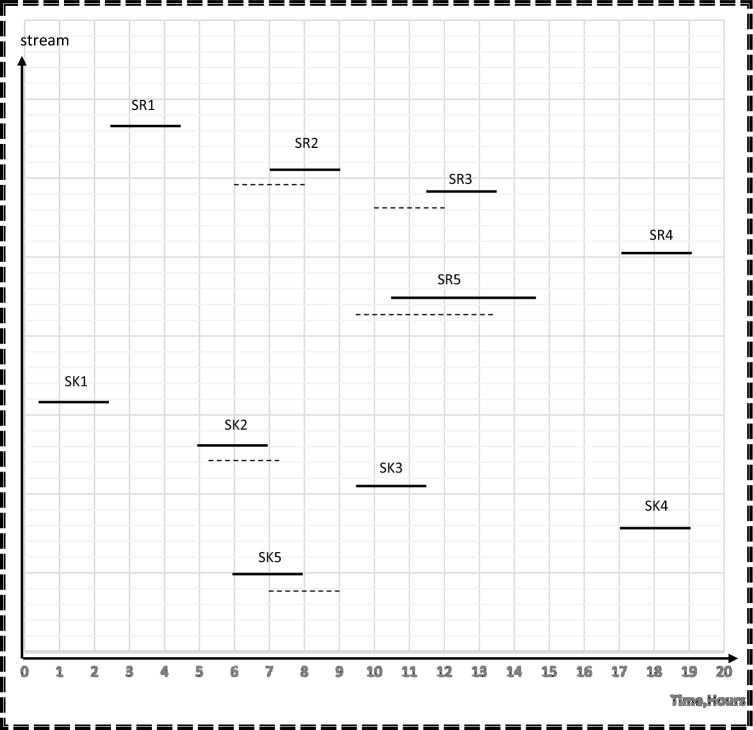
Time dependent chart for case study 1 after rescheduling.

**Figure 7:**
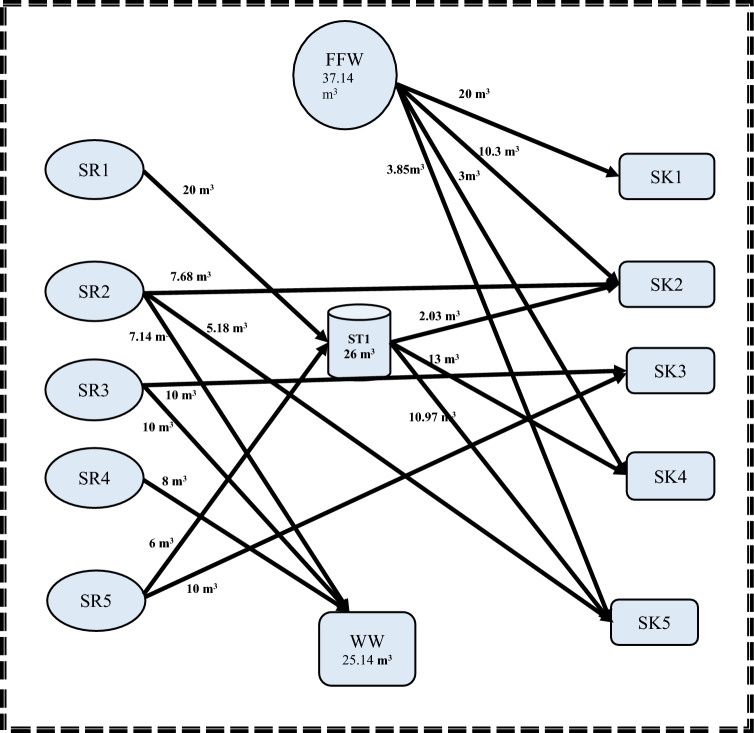
Batch water network after rescheduling for case study 1.

The applied model also shows the optimal degree of streams’ shifting that achieved the minimum cost without a high schedule violation. A high schedule violation, characterized by a substantial deviation in the commencement and completion times of a process in comparison to the primary schedule, is considered unfavorable within the scope of this study, since high schedule violation may negatively affect the process operation. Additionally, such deviations have the potential to yield an impractical network configuration. In the context of rescheduling for direct reuse, such violations can disrupt the project’s timeline, impacting resource allocation, quality control, stakeholder satisfaction, project risk, and contractual obligations. Effective project management is essential for minimizing these adverse effects. As shown in Fig. 6, duration of source streams; SR1 and SR4 and sink streams; SK1, SK3 and SK4 remain constant. The durations of source streams SR2 and SR5 are shifted before its time by 1 h but the starting and ending time of SR3 is shifted by 1.5 h before its table time. That is why 1.5 h is considered the maximum shifting value that occurs in this model. The duration of sink stream SK5 is shifted by 1h after its table time, but sink stream SK2 is shifted after its table time by 0.25 h. Consequently, 0.25 h is taken as the minimum shifting value. Sources and sinks are represented in their corresponding time intervals as continuous lines before rescheduling and as discontinuous ones which show the opportunities for shifting after rescheduling so as to maximize direct water recycle/reuse. The water network after rescheduling is shown in Fig. 7.

Regarding case study 2, equations of step 1 are applied with 17 constraints and 26 linear variables in the entire mathematical formulation. The resulted minimum freshwater flow and wastewater discharge are 70 and 50 tons, respectively.

**Figure 8:**
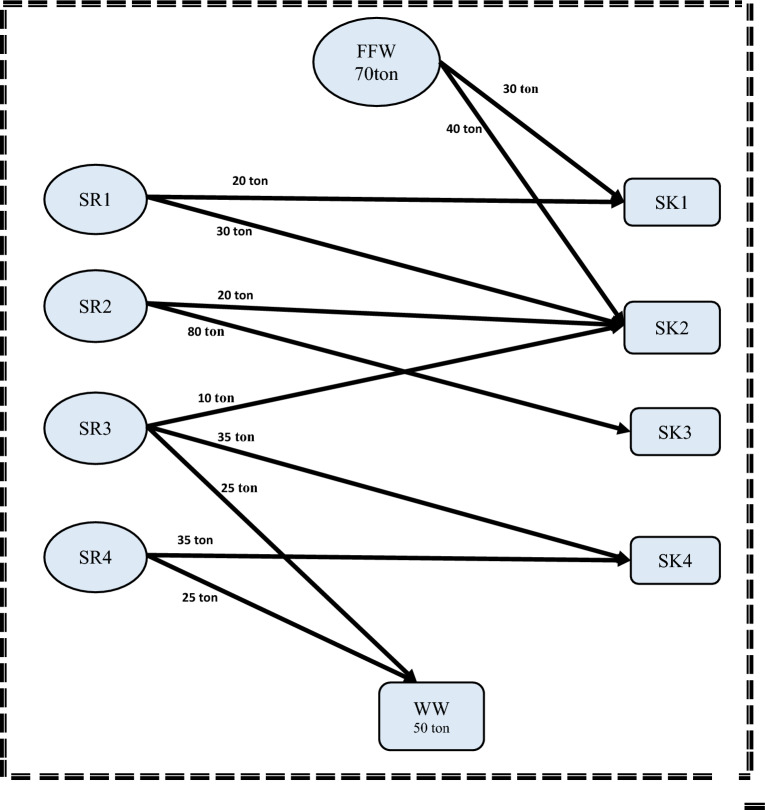
Water network with minimum freshwater for case study 2.

**Figure 9:**
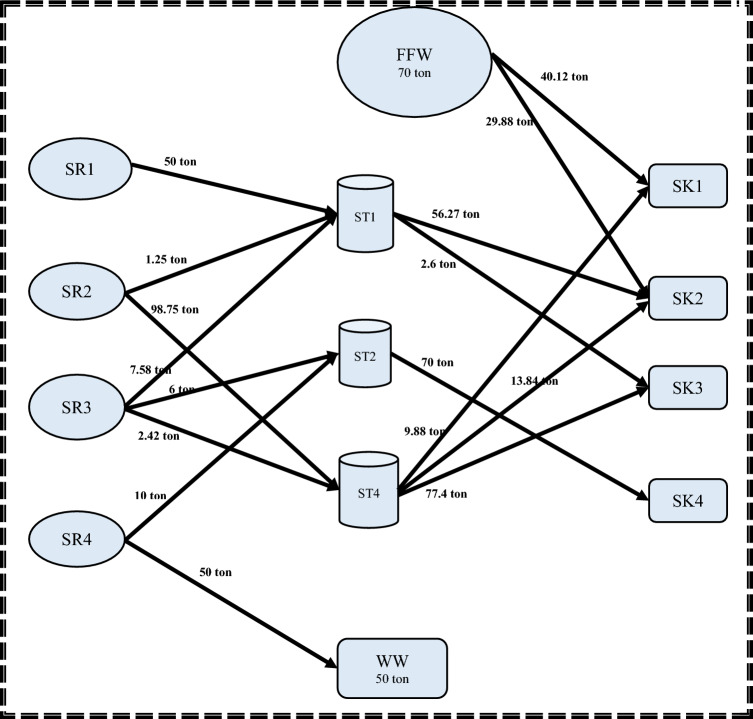
Water network with minimum number of tanks for case study 2.

Considering step 2, the model has 65 variables, 24 non-linear variables, and 4 integers with 33 constraints in its MINLP mathematical formulation. The computational process for this step, from initiation to completion, took 13 s. The model results show that there are three minimum storage tanks with a total capacity of 230 tons. Storage 1 (FST_1_ = 58.839 ton; CST_1_ = 63.957 ppm), Storage 2 (FST_2_ = 70 ton; CST_2_ = 164.2857 ppm) and storage 4 (FST_4_ = 101.161 ton; CST_4_ = 101.1934 ppm). It is worth noting that the water network meets the minimum fresh and wastewater flow targets established in step 1. These results matched well with the results obtained in Shoaib et al. work^4^. The mathematical results for steps 1and 2 are shown in Figs. 8 and 9 respectively.

In the third stage, the results from stages 1 and 2 will be used as aspiration levels of Z_n_(x) for freshwater and number of tanks functions. Also, time dependent chart is used as explained in case study1. For example, minimum freshwater from step 1, which is 70 m^3^, is the aspiration Level for Z_1_(x) for freshwater function. The goal programming model has 52 variables, 24 non-linear variables, and 46 constraints in its mathematical formulation. The computational process for this model, which includes solving and obtaining results, took 24 s.

Comparing the results of water network after and before rescheduling for the second case study, the flowrate of freshwater has been decreased to be 150 ton whereas the freshwater flowrate before rescheduling was 191.825 ton. Moreover, the wastewater flowrate has been decreased from 171.825 ton to 130 ton. The capacity of tank used before rescheduling is 58.2 ton (with concentration CST1 = 207.2 ppm). The results show also that the number of storage tanks required is reduced from 1 to zero as shown in Table 5.

**Table 5:** Comparison of the results before and after rescheduling for case 2.

Parameters	Before rescheduling	After rescheduling	Percentage of decreasing (%)
Freshwater	191.825 ton	150 ton	21.8
Wastewater	171.825 ton	130 ton	24.4
Number of tanks	1	0	100
Capacity of tanks	58.2 ton	0	100
Cost	224,276.1 $	169,281.4 $	24.6

According to the economic study, the total cost required for the network after rescheduling is reduced to $169,281.4 compared to $224,276.1 before rescheduling with a saving percent of 24.6%. This saving can be attributed to the non- existence of storage tanks and the reduction of the freshwater flowrate by 21.8%.

**Figure 10:**
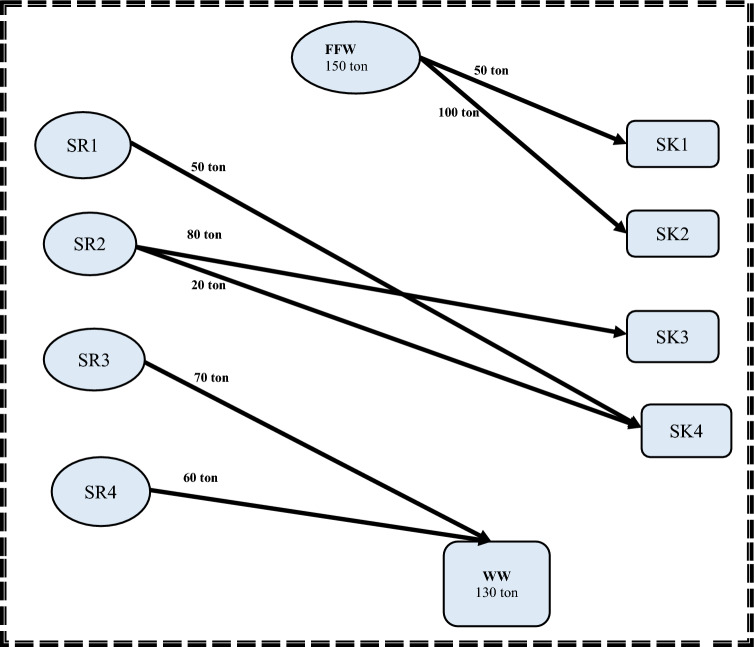
Batch water network after rescheduling for case study.

**Figure11:**
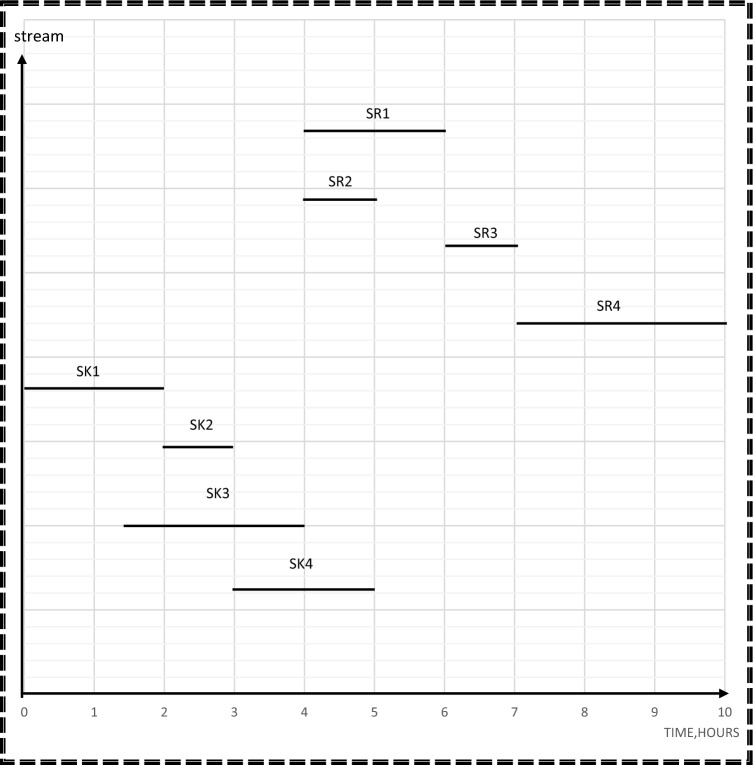
Time dependent chart for case study 2 before rescheduling.

**Figure12:**
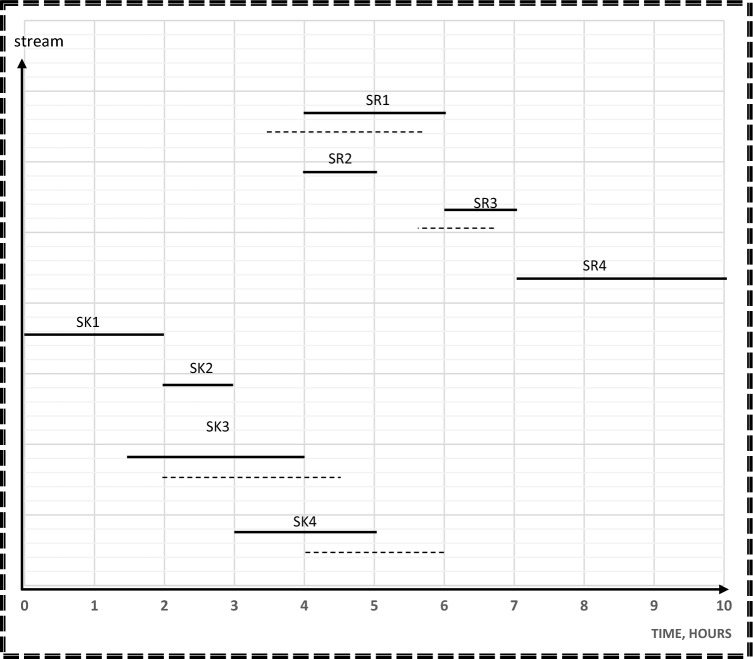
Time dependent chart for case study 2 after rescheduling.

The water network after rescheduling for this case study is shown in Fig. 10. The introduced model also shows the optimal degree of streams’ shifting that achieved the minimum cost without a high schedule violation. The duration of SR2 and SR4 as source streams and sink streams SK1 and SK2 remain constant. The durations of source streams SR1 and SR3 are shifted before their table time by 0.5h. Therefore, the value of 0.5 h is considered as the minimum shifting value. The duration of sink stream SK3 is shifted by 0.5h after its table time, but sink stream SK4 is shifted after its table time by 1h. Thus, the value of 1 h is considered as the maximum shifting value used in this model. Time dependent charts before and after rescheduling for the case study 2 are shown in Figs. 11 and 12 respectively.

Within the framework of Case Study 3, focused on a multi-contaminant batch water network, the application of equations in Step 1 involves 21 constraints and 26 linear variables. This results in a minimum freshwater flow of 70 tons and a wastewater discharge of 50 tons.

Transitioning to Step 2, the model comprises 57 variables, including 28 non-linear variables and 4 integers, with 39 constraints in its MINLP mathematical formulation. The computational process for this step, from initiation to completion, concluded in 12 s. Model results indicate the existence of four minimum storage tanks with a total capacity of 230 tons: Storage 1 (FST1 = 69.05 ton; CST_a1_ = 150 ppm; CST_b1_ = 120 ppm), Storage 2 (FST2 = 10 ton; CST_a2_ = 250 ppm; CST_b2_ = 240 ppm), Storage 3 (FST3 = 88.82 ton; CST_a3_ = 72.1 ppm; CST_b3_ = 39 ppm), and storage 4 (FST4 = 62.13 ton; CST_a4_ = 100.74 ppm; CST_b4_ = 50.66 ppm). It is noteworthy that the water network meets the minimum fresh and wastewater flow targets established in step 1.

In the third stage, the results from stages 1 and 2 serve as aspiration levels for Zn(x) in the freshwater and number of tanks functions. Additionally, a time-dependent chart is utilized. The goal programming model comprises 59 variables, including 32 non-linear variables, and aligns with 54 constraints in its mathematical formulation. The computational process, entailing solving and obtaining results for this model, took 24 s.

Table 6 illustrates the comparison between water network results before and after rescheduling for the third case study. The freshwater flowrate has decreased to 110 tons, contrasting with the pre-rescheduling value of 148.1 tons. Additionally, the wastewater flowrate has been reduced from 128.1 tons to 90 tons. The results also reveal a decrease in the required number of storage tanks from 4 to 1. The tank has a capacity of 141 tons (with concentrations of CT_a1_ = 113.5 ppm and CT_b1_ = 69.5 ppm).

**Table 6:** Comparison of the results before and after rescheduling for case 2.

Parameters	Before rescheduling	After rescheduling	Percentage of decreasing (%)
Freshwater	148.1 ton	110 ton	25.7
Wastewater	128.1 ton	90 ton	29.7
Number of tanks	4	1	75
Cost	190,720.3 $	161,874.8 $	15.1

In economic terms, the network’s total cost post-rescheduling has significantly decreased to $161,874.8, a noteworthy reduction from the pre-rescheduling cost of $190,720.3. This translates to a savings percentage of 15.1%. The key driver behind this cost reduction is the significant 25.7% reduction in the flowrate of freshwater.

**Figure13:**
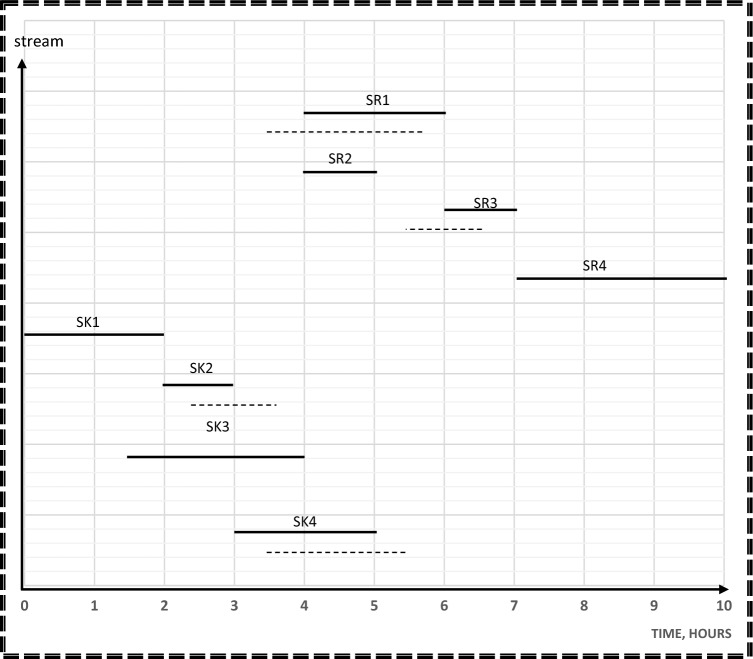
Time dependent chart for case study 3 after rescheduling.

The presented model shows the optimal degree of streams’ shifting, achieved the minimum cost without a high schedule violation. The duration of SR2 and SR4 as source streams and sink streams SK1 and SK3 remain constant. For source streams SR1 and SR3, their durations are shifted 0.5 h earlier than their scheduled time. Similarly, the duration of sink streams SK2 and SK4 is shifted by 0.5 h after their scheduled. Figure 13 show the time-dependent chart after rescheduling for case study 3.

## Conclusion

This study develops a goal programming approach that is used for batch water process rescheduling by applying LINGO software. The introduced model presents a novel mixed integer nonlinear program (MINLP) for multi-objective optimization. This multi objectives model seeks to achieve the required freshwater, wastewater, number and capacity of tanks, minimum degree of shifting streams, as well as the minimum total cost. Three case studies were investigated in this work to demonstrate the effectiveness of the applied technique for water network rescheduling, considering both single and multi-contaminants problems. The results reveal an observable reduction in the capacity of storage tanks and consequently a reduction in the network total cost with an optimal degree of stream shifting and without high schedule violation. Rescheduling of the batch water network of the first case study leads to reducing the total network cost by 26.4% and reducing the storage tank capacity by 18.8%. Regarding the second case study, the water network rescheduling results in reducing the network total cost by 24.6% and decreasing the freshwater flowrate by 21.8% without the need of storage tanks. The third case study on multi-contaminants batch water network results in a 15.1% cost reduction and a 25.7% decrease in freshwater consumption. These results show that the introduced technique used for batch water networks rescheduling is effective and increases the economic benefits of the investigated networks. Therefore, the introduced rescheduling method can be applied for other batch water networks to increase their profits.

## Data Availability

All data generated or analyzed during this study are included in this published article.

## List of symbols

$${C}_{I}$$: Cost of interception ($)
$${C}_{FW}$$: Cost of fresh water ($)
$${C}_{SKj}$$: Maximum inlet impurity concentration to sink *j* (ppm)
$${C}_{SRi}$$: Impurity concentration of source *i* (ppm)
$${C}_{STk}$$: Impurity concentration of storage tank *k* (ppm)
$$C{T}_{a}$$: Impurity concentration for contaminant a (ppm)
$$C{T}_{b}$$: Impurity concentration for contaminant b (ppm)
$${C}_{ST}$$: Cost of storage tanks ($)
$${F}_{Fw}$$: Total freshwater resource fed to the water network (ton or m^3^)
$${F}_{FW}{,}_{SKj}$$: Amount of flow from freshwater resource going to sink *j* (ton or m^3^)
$${F}_{SKj}$$: Water flow demand for sink *j* (ton or m^3^)
$${F}_{SRi}$$: Water flow of source *i* (ton or m^3^)
$${F}_{STk}$$: Capacity of storage tank *k* (ton or m^3^)
$${F}_{SRi}{,}_{SKj}$$: Amount of flow from water resource going to sink *j* (ton or m^3^)
$${F}_{SRi}{,}_{STk}$$: Amount of flow from source *i* going to storage tank *k* (ton or m^3^)
$${F}_{STk}{,}_{SKj}$$: Amount of flow from storage tank *k* going to sink *j* (ton or m^3^)
$${F}_{WW}$$: Total amount of flow going to waste (ton or m^3^)
$${F}_{WW}{,}_{SRi}$$: Wastewater discharged from source *I* (ton or m^3^)
$${G}_{n}$$: The aspiration level of nth goal
$${I}_{k}$$: A binary variable denotes the existence of storage *k*
$${N}_{k}$$: Total number of water storage tanks
$$r$$: Parameter of storage tank cost: slope of linear relationship
$$s$$: Parameter of storage tank cost: interception of linear relationship
$${U}_{k}$$: Maximum permitted capacity of the storage tank
$${Z}_{n}$$: Linear function of nth goal
